# Evaluation of Peer-to-Peer Support and Health Care Utilization Among Community-Dwelling Older Adults

**DOI:** 10.1001/jamanetworkopen.2020.30090

**Published:** 2020-12-15

**Authors:** Elizabeth A. Jacobs, Rebecca Schwei, Scott Hetzel, Jane Mahoney, Katherine Sebastian, Kali DeYoung, Jenni Frumer, Jenny Madlof, Alis Simpson, Erika Zambrano-Morales, KyungMann Kim

**Affiliations:** 1 Department of Medicine, University of Texas at Austin Dell Medical School, Austin; 2 Department of Population Health, University of Texas at Austin Dell Medical School, Austin; 3 Now with Maine Medical Center Research Institute, Scarborough; 4 Department of Emergency Medicine, University of Wisconsin School of Medicine and Public Health, Madison; 5 Department of Biostatistics and Biomedical Informatics, University of Wisconsin School of Medicine and Public Health, Madison; 6 Department of Medicine, University of Wisconsin School of Medicine and Public Health, Madison; 7 BioTel Research, Rockville, Maryland; 8 Jenni Frumer & Associates, LLC, West Palm Beach, Florida; 9 Alpert Jewish Family Service of West Palm Beach, West Palm Beach, Florida; 10 Department of Higher Education and Human Development, University of Rochester, Rochester, New York; 11 Department of Psychology, California State University, Los Angeles

## Abstract

**Importance:**

The vast majority of older adults desire to age in their communities, and it is not clear what helps them be successful at aging in place.

**Objective:**

To investigate the comparative effectiveness of community-designed and community-implemented peer-to-peer (P2P) support programs vs standard community services (SCS) to promote health and wellness in at-risk older adults.

**Design, Setting, and Participants:**

This comparative effectiveness study involved a longitudinal cohort of adults aged 65 years and older conducted between 2015 and 2017. The setting was 3 communities in which community-based organizations delivered P2P services to older adults in California, Florida, and New York. Participants in the P2P group and in the SCS group were matched at enrollment into the study according to age, sex, and race/ethnicity at each site. Data analysis was performed from October 2018 to May 2020.

**Exposures:**

P2P support was provided by trained older adult volunteers in the same community. They provided support targeted at the needs of the older adult they served, including transportation assistance, check-in calls, social activities, help with shopping, and trips to medical appointments.

**Main Outcomes and Measures:**

Rates of hospitalization, urgent care (UC) and emergency department (ED) use, and a composite measure of health care utilization were collected over 12 months of follow-up.

**Results:**

A total of 503 participants were screened, 456 participants were enrolled and had baseline data (234 in the SCS group and 222 in the P2P group), and 8 participants had no follow-up data, leaving 448 participants for the main analysis (231 in the SCS group and 217 in the P2P group; 363 women [81%]; mean [SD] age, 80 [9] years). Participants in the P2P group more often lived alone, had lower incomes, and were more physically and mentally frail at baseline compared with the SCS group. After adjusting for propensity scores to account for baseline differences between the 2 groups, there was a statistically significant higher rate of hospitalization in the P2P group than in the SCS group (0.68 hospitalization per year vs 0.44 hospitalization per year; risk ratio, 1.54; 95% CI, 1.14-2.07; *P* = .005) during the 12 months of observation. There were no significant differences between the 2 groups in the rates of ED or UC visits or composite health care utilization over the 12 months of the study.

**Conclusions and Relevance:**

P2P support was associated with higher rates of hospitalization but was not associated with other measures of health care utilization. Given that this is not a randomized clinical trial, it is not clear from these findings whether peer support will help older adults age in place, and the topic deserves further study.

## Key Points

**Question:**

Is a community-designed and community-implemented peer-to-peer (P2P) support program for older adults associated with lower acute health care utilization?

**Findings:**

In this comparative effectiveness study, there was a statistically significant higher rate of hospitalization in the P2P group than the standard community services group during the 12 months of observation. There were no significant differences in the rates of emergency department and urgent care visits or in the composite outcome of any health care utilization between the 2 groups.

**Meaning:**

These findings suggest that P2P support programs may change the way in which older adults access health care and lead to greater hospitalization.

## Teaser

This comparative effectiveness study investigates whether a community-designed peer-to-peer support program is associated with lower acute health care utilization compared with standard community services among older adults.

## Introduction

In 2010, 40 million people aged 65 years and older lived in the US, accounting for 13% of the total population.^1^ The older population is projected to be twice as large in 2030, growing from 35 million to 72 million and representing 20% of the total US population.^1^

Most people aged 65 years and older report their health as good, very good, or excellent, but with age comes increased risk of certain diseases and disorders.^1^ These health conditions and the process of aging itself frequently lead to functional decline. In 2013, approximately 44% of people aged 65 years and older enrolled in Medicare reported a functional limitation; approximately 28% had difficulty with at least 1 activity of daily living (ADL), such as eating, bathing, dressing, using the restroom independently, and walking.^2^

Functional decline puts older adults at risk for entering into nursing homes, yet more than 90% of older adults want to continue to age in place, defined as “… being able to remain in one’s current residence even when faced with increasing need for support.”^3,4,5^ The question of how to effectively help older adults age in place is of great national importance given the aging of the US population, the high prevalence of functional limitations among older adults, and this at-risk population’s overwhelming desire to age in their own homes.

Despite the importance of this question, most of the research has focused on what factors place older adults at risk of moving from community living to a nursing home rather than what can be done to prevent this transition.^6,7^ Although this research helps us understand what factors should be targeted by interventions to promote aging in place, it does not provide evidence for what we can do to prevent nursing home placement.

Peer-to-peer (P2P) support is a community-based service that may support aging in place.^8^ These programs promote social relationships and engagement with services through interaction with a peer who helps with accessing social opportunities, health care, and other services within the community.

We examined the effectiveness of P2P community support in preventing factors known to be associated with moving out of the community—frequent health care utilization measured by hospitalization and urgent care (UC) and emergency department (ED) use—in an at-risk older adult population compared with a standard community services (SCS) group. We hypothesized that older adults in the P2P group would have lower rates of hospitalizations and UC and ED visits compared with the SCS group.

## Methods

We conducted an observational, longitudinal, comparative effectiveness study in 3 communities in the US: Los Angeles, California; Rochester, New York; and West Palm Beach, Florida.^9^ The community-based organizations with which we partnered serve racially and ethnically diverse older adults. The Western Institutional Review Board approved the study protocol and informed consent documents; all participants provided written informed consent. We followed the International Society for Pharmacoeconomics and Outcomes Research (ISPOR) reporting guideline for reporting comparative effectiveness research.^10^

### Participants

We enrolled older adults and matched participants in the SCS and P2P groups at each study site according to age (<70, 70-79, and ≥80 years), sex (male and female), and race/ethnicity (African American or Black, Asian, White, and other; Hispanic and non-Hispanic) on the basis of the statistical distribution of these demographic characteristics of the older adults receiving P2P support at each site. We chose to match on these characteristics because age, sex, and race/ethnicity are factors associated with successful aging in place.^7^ We periodically reviewed our matching and targeted recruitment to ensure adequate matching on each characteristic in the 2 study groups. At each site, we recruited potential participants from the P2P support programs and sites receiving SCS by the organizations also providing the P2P support programs.

To be included in the study, older adults had to be aged 65 years or older, live independently in their community year-round, and meet the community-defined criteria for receiving P2P support, including living at or below poverty level or on a fixed income that made meeting their living expenses challenging, being socially isolated, having chronic illnesses, or frequently using community services or resources that the organization offered. During eligibility screening, we asked participants whether they lived independently with or without family, in independent care, in assisted living, or in a nursing home. For this study, independent care was defined as a type of residence designed for seniors who require little or no assistance with the ADLs, although services such as housekeeping, laundry, and meals may be provided. In addition, some home health care services may be provided by in-house staff or an outside agency. We excluded older adults who were receiving care for 40 hours or more per week from a caregiver such as a home health aide; adults who were younger than 65 years, lived in assisted living or a nursing home, or were planning on moving or entering long-term care in the next 12 months; and adults with cognitive impairment, which was defined as having a score of 25 or less on the Telephone Interview for Cognitive Status instrument.^11^

To be included in the P2P group, older adults also had to be enrolled in this program within their community and to have an assigned peer volunteer. Some participants were newly enrolled in the P2P program; most had been participating in the program for many years. To be included in the SCS group, older adults had to meet the qualifications for receiving P2P support from the community organization but not be enrolled in P2P support.

### Intervention: P2P Services

All 3 study sites had P2P programs with standardized core elements, as outlined in Table 1, including a similar definition of who qualifies for P2P support. Peers had to be aged 55 years or older, and most received small stipends for being volunteers. Older adults in the P2P support programs continued to have access to standard services even while they were enrolled in the P2P group.

**Table 1.** Core Elements of the Peer-to-Peer Community Support Intervention

Element	Description
Goal	To promote successful aging in place among frail older adults
Duration of peer-to-peer relationship	From enrollment until patient transitioned to more advanced care, left the area, or died
Target population	Older adults at risk for a decline in health or placement in long-term care
Referral process	Self-referral or referral by case managers from the community organization or local health care organizations
Volunteer selection	Adults aged >65 y who were able to dedicate 20 h/wk to peer support Needed to commit for a minimum of 1 y
Volunteer training	Initial training: 10-20 h Training modules Developing a peer-to-peer support relationship Importance of companionship Basic health and emotional health needs of at-risk older adults How to provide emotional support An overview of services provided by the organization and by the community and how to access them Trouble-shooting particular issues that might arise in a relationship Monthly in-service training: 1-2 h on a relevant topic
Expectations of volunteers	Attend all trainings Attend at least 60% of monthly in-service trainings Provide a minimum of 20 h of peer support/mo Contact assigned peers on a regular basis
Peer client load per volunteer	Minimum of 2 to a maximum of 10 peer clients
Shaped to meet the local community needs	Each program added training on particular issues or community resources unique to the community they serve

Older adults in the SCS group had access to all the services at each participating community organization provided, with the exception of P2P support. The standard aging services offered at each site included such items as health, wellness, socialization, and enrichment activities; case management and counseling; resource referrals; food pantry assistance; and meal delivery.

### Outcomes

We compared the rate of UC, ED, and hospital utilization and a composite measure of all 3 in the group receiving P2P support with that of the group receiving SCS. We collected data on the number of UC, ED, and hospital visits at 4 different time points for each individual: 3, 6, 9, and 12 months. We asked how many times, over the preceding 3-month period, respondents had visited a UC clinic or an ED or stayed in the hospital overnight or longer. We asked additional questions about the dates of health care use and names of places where they sought care. These questions have been shown to have high test-retest reliability in an older population of individuals with chronic disease and are correlated with actual documented usage.^12^ To facilitate recall of this information, we provided each participant with a preprinted study calendar on which they could circle *UC* for urgent care, *ED* for emergency department, or *HOSP* if they stayed overnight in the hospital. We asked participants to refer to this calendar when being surveyed.

Because recall bias may affect the validity of these data, we asked participants for permission to obtain their medical records. We then validated the data by randomly selecting 10% of participants in each group at each site and reviewed their reported use with the documentation in their medical records. This technique is frequently used when recall bias poses a data problem to get a picture of the reliability of the recalled data.^13^

### Additional Variables

We collected data on the following sociodemographic characteristics: age (in years), race and ethnicity (African American or Black, Asian, Hispanic, White, or other), and marital status (never married, married, widowed, separated, or divorced). In addition, we asked about current living arrangements (alone, with partner, with other family member, and/or other caregiver), current income and total household income, and years of formal schooling completed. We also documented in which language the surveys were completed (English or Spanish). In addition, to control for functional and mental health, we collected data on functional status using the 12-Item Short Form Survey, depressive symptoms using the Center for Epidemiological Studies Depression, anxiety using the Geriatric Anxiety Inventory–Short Form, loneliness using the Short Scale for Measuring Loneliness in Large Surveys, efficacy using the General Self Efficacy Scale, resilience using the Brief Resilience Scale, and social support and self-reported disability and physical function using the ADLs and instrumental ADLs scales.^14,15,16,17,18,19,20,21,22,23^

### Statistical Analysis

We compared the baseline participant characteristics using 2-sample *t* test, Wilcoxon rank sum test, Fisher exact test, and χ^2^ test, depending on the variable, at a 2-tailed significance level of *P* < .05 without adjustment for multiplicity of outcomes and testing. The baseline characteristics, other than age, sex, and race/ethnicity, of the 2 groups were not similar (Table 2) so we adjusted for these differences using a propensity score for each participant. We conducted a logistic regression analysis using intervention group status (P2P vs SCS) as the outcome and baseline characteristics as covariates to understand the association of group status with baseline measures of previous health care utilization, health status, well-being, and demographic information. We included baseline patient characteristics that were associated with being in the P2P group at the *P* < .15 level to estimate a propensity score for each participant. We weighted our final regression models using the inverse propensity score for each participant.^24^ Variables included in the propensity score can be found in the eTable in the Supplement.

**Table 2.** Study Sample Characteristics by Group and by Site

Variables	Overall			Los Angeles, CA			Rochester, NY			West Palm Beach, FL		
	SCS (n = 231)	P2P (n = 217)	*P* value	SCS (n = 40)	P2P (n = 35)	*P* value	SCS (n = 100)	P2P (n = 96)	*P* value	SCS (n = 91)	P2P (n = 86)	*P* value
Demographic variables, participants, No. (%)												
Site												
Los Angeles	91 (39.4)	86 (39.6)		40 (100.0)	35 (100.0)		0	0		0	0	
Rochester	100 (43.3)	96 (44.2)		0	0		100 (100.0)	96 (100.0)		0	0	
West Palm Beach	40 (17.3)	35 (16.1)		0	0		0	0		91 (100.0)	86 (100.0)	
Female	183 (79.2)	180 (82.9)	.38	27 (67.5)	25 (71.4)	.91	85 (85.0)	87 (90.6)	.33	71 (78.0)	68 (79.1)	>.99
Race/ethnicity												
African American	24 (10.4)	23 (10.6)	.81	1 (2.5)	1 (2.9)	.61	23 (23.0)	22 (22.9)	.70	0	0	>.99
Asian	2 (0.9)	2 (0.9)		1 (2.5)	2 (5.7)		1 (1.0)	0		0	0	
Hispanic	23 (10.0)	20 (9.2)		6 (15.0)	2 (5.7)		16 (16.0)	17 (17.7)		1 (1.1)	1 (1.1)	
White	182 (78.8)	170 (78.3)		32 (80.0)	30 (85.7)		60 (60.0)	55 (57.3)		90 (98.9)	85 (98.8)	
Other	0	2 (0.9)		0	0		0	2 (2.1)		0	0	
Age, mean (SD), y	79.2 (8.7)	80.5 (9.3)	.13	77.4 (8.3)	81.0 (8.9)	.08	76.1 (8.0)	76.8 (8.7)	.57	83.3 (7.9)	84.4 (8.6)	.40
Marital status												
Never married	18 (7.9)	24 (10.9)	.04	8 (20.5)	4 (11.4)	.09	8 (8.1)	13 (13.1)	.56	2 (2.2)	7 (8.0)	.005
Married	51 (22.3)	22 (10.0)		7 (17.9)	1 (2.9)		17 (17.2)	12 (12.1)		27 (29.7)	9 (10.3)	
Widowed	100 (43.7)	112 (50.7)		12 (30.8)	17 (48.6)		42 (42.4)	40 (40.4)		46 (50.5)	55 (63.2)	
Divorced or separated	60 (26.2)	63 (28.5)		12 (30.8)	13 (37.1)		32 (32.3)	34 (34.3)		16 (17.6)	16 (18.4)	
Lives alone	162 (69.8)	177 (79.7)	.02	30 (76.9)	25 (71.4)	.78	70 (68.6)	77 (77.0)	.24	62 (68.1)	75 (86.2)	.007
Total household income, $												
<10 000	20 (11.7)	35 (21.3)	<.001	7 (21.2)	5 (16.7)	.11	12 (13.0)	22 (24.2)	.02	1 (2.2)	8 (18.6)	.008
10 000-14 999	50 (29.2)	61 (37.2)		10 (30.3)	18 (60.0)		32 (34.8)	34 (37.4)		8 (17.4)	9 (20.9)	
15 000-24 999	47 (27.5)	48 (29.3)		12 (36.4)	6 (20.0)		24 (26.1)	26 (28.6)		11 (23.9)	16 (37.2)	
25 000-49 999	44 (25.7)	15 (9.1)		4 (12.1)	1 (3.3)		21 (22.8)	6 (6.6)		19 (41.3)	8 (18.6)	
≥50 000	10 (5.8)	5 (3.0)		0	0		3 (3.3)	3 (3.3)		7 (15.2)	2 (4.7)	
Years of schooling	13.5 (3.2)	13.1 (3.5)	.21	13.4 (4.1)	13.6 (3.3)	.76	13.3 (3.5)	12.7 (3.8)	.27	13.8 (2.2)	13.3 (3.3)	.27
Spanish language preference	7 (3.9)	12 (6.2)	.42	3 (7.5)	1 (2.9)	.71	4 (4.3)	11 (13.8)	.05	0	0	NA
Health, loneliness, self-efficacy, resilience, and social support measures at baseline												
Health status and quality of life score, mean (SD)												
Mental health	52.2 (12.7)	49.3 (13.3)	.02	46.5 (14.0)	49.8 (14.2)	.33	54.3 (12.0)	51.0 (11.7)	.05	52.3 (12.2)	47.0 (14.5)	.01
Physical health	41.3 (12.7)	36.1 (13.0)	<.001	39.9 (13.7)	31.1 (11.9)	.004	39.3 (12.6)	38.4 (13.2)	.63	44.0 (11.9)	35.5 (12.8)	<.001
Depressive symptoms	3.6 (1.8)	3.9 (1.9)	.07	3.8 (2.0)	3.9 (1.5)	.88	3.6 (1.7)	4.0 (1.9)	.15	3.5 (1.7)	3.9 (2.0)	.20
Anxiety symptoms	1.4 (1.7)	1.8 (1.8)	.02	1.9 (1.8)	1.9 (1.9)	.92	1.4 (1.6)	1.6 (1.7)	.29	1.2 (1.6)	1.9 (1.9)	.01
Loneliness	1.5 (0.6)	1.7 (0.7)	<.001	1.8 (0.7)	1.8 (0.7)	.87	1.6 (0.6)	1.6 (0.6)	.83	1.3 (0.5)	1.8 (0.7)	<.001
Self-efficacy	3.4 (0.5)	3.2 (0.6)	<.001	3.4 (0.5)	3.2 (0.6)	.17	3.4 (0.5)	3.2 (0.6)	.12	3.5 (0.6)	3.2 (0.7)	<.001
Resilience	3.6 (0.9)	3.4 (0.8)	.08	3.2 (1.1)	3.4 (1.0)	.30	3.6 (0.7)	3.5 (0.8)	.20	3.7 (0.8)	3.4 (0.8)	.01
Social support	3.7 (1.2)	3.6 (1.0)	.23	2.9 (1.3)	3.5 (1.0)	.03	3.8 (1.1)	3.6 (1.0)	.17	3.9 (1.1)	3.6 (1.0)	.03
Self-reported disability												
ADLs, median (IQR)	0.0 (0.0-0.0)	0.0 (0.0-1.0)	<.001	0.0 (0.0-0.0)	0.0 (0.0-1.0)	.05	0.0 (0.0-0.0)	0.0 (0.0-1.0)	.10	0.0 (0.0-0.0)	0.0 (0.0-1.0)	.01
Instrumental ADLs, median (IQR)	8.0 (7.0-8.0)	7.0 (5.0-8.0)	<.001	8.0 (7.0-8.0)	6.0 (5.0-7.0)	<.001	7.0 (6.0-8.0)	7.0 (6.0-7.0)	.006	8.0 (8.0-8.0)	7.0 (6.0-8.0)	<.001
Mobility/strength, mean (SD)	1.3 (1.0)	1.9 (1.0)	<.001	1.2 (0.9)	2.2 (0.8)	<.001	1.4 (1.1)	1.6 (1.1)	.21	1.3 (1.0)	2.0 (1.0)	<.001
Physical function, mean (SD)	2.3 (1.5)	3.1 (1.4)	<.001	2.5 (1.7)	3.1 (1.4)	.07	2.6 (1.4)	3.0 (1.5)	.07	1.9 (1.4)	3.1 (1.4)	<.001

Abbreviations: ADLs, activities of daily living; IQR, interquartile range; P2P, peer-to-peer per group; SCS, standard community services.

Specifically, we performed a negative binomial regression analysis with the inverse propensity score weighting for count outcomes with site as a covariate and days in the study as an offset to estimate the annualized rate of the number of UC visits, ED visits, and hospitalizations and to estimate the risk ratio (RR) between the P2P and SCS groups. We used the same method for conducting logistic regression analysis for the binary outcome of any UC visits or ED visits.

We had missing data because of dropout of participants, including 8 participants who only had baseline data, and because participants died or transitioned to a higher level of care before month 12. As a sensitivity analysis and to account for the 35 dropouts (7.7%), we compared the baseline characteristics of the dropouts and the participants who completed the study; those who dropped out were mostly Hispanic women from the Rochester site. We performed the same propensity score analysis of the outcomes with and without the dropouts, and there was no difference in our findings. As another sensitivity analysis, we performed the same propensity score analysis including the death and transition to a higher level of care as part of the composite primary outcome; our findings were the same in this sensitivity analysis as in the main analysis. Data analysis was performed from October 2018 to May 2020 using R statistical software version 3.5.1 (R Project for Statistical Computing).

## Results

**Figure 1.**
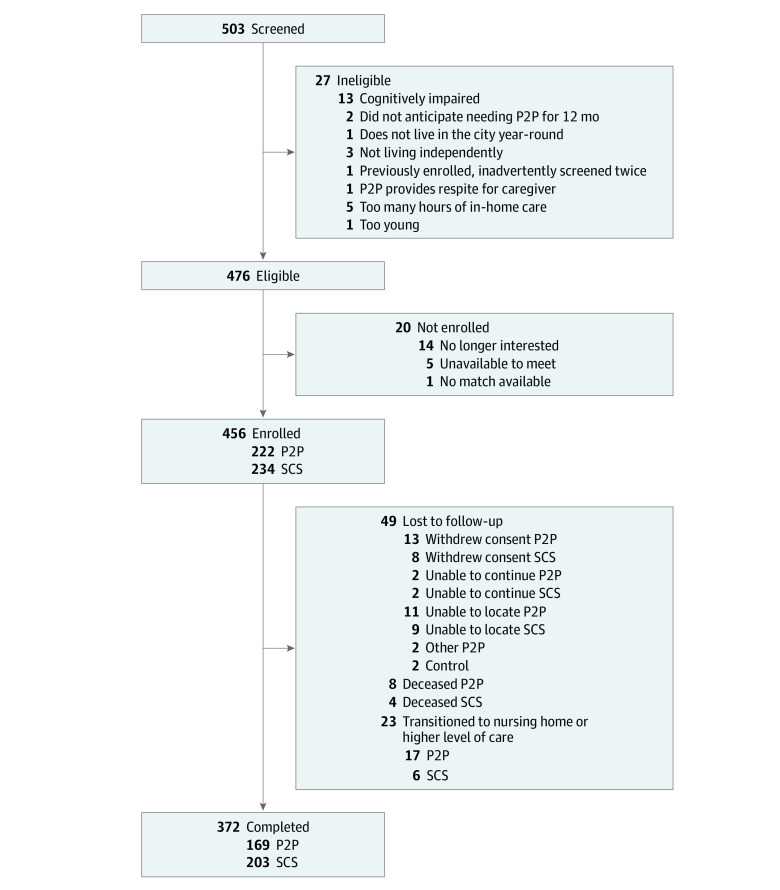
Enrollment Flowchart for the Aging in Place Study Participants P2P indicates peer-to-peer; and SCS, standard community services.

We screened 503 older adults for inclusion in our study (Figure 1). Twenty-seven participants were ineligible, and 20 eligible participants were not enrolled, leaving 456 adults in our final sample: 234 in the SCS group and 222 in the P2P group. Eight participants had no follow-up data, leaving 448 participants, including 231 in the SCS group and 217 in the P2P group, for the main analysis.

**Figure 2.**
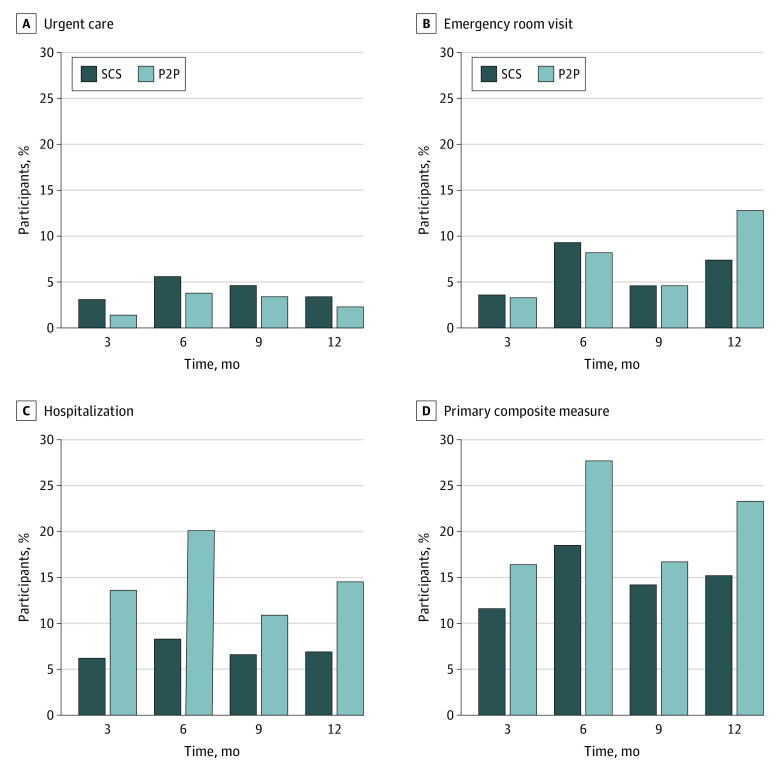
Percentage of Peer-to-Peer (P2P) and Standard Community Services (SCS) Participants Who Visited an Emergency Department or Urgent Care or Were Hospitalized at 3, 6, 9, and 12 Months

Most of the participants in the study were women (363 women [81%]) and self-identified as White (352 individuals [78%]) (Table 2). The mean (SD) age of participants was 80 (9) years, and 19 participants (5%) preferred to conduct the study in Spanish. The 2 study groups were balanced within each site by the matching characteristics: age, sex, and race/ethnicity. Several baseline measures differed significantly by group: the P2P group more often lived alone, had lower income, were more anxious, were lonelier, and had worse self-efficacy and physical functioning than the SCS group at baseline (Table 2). The use of health care was high in both groups, with 101 participants (40%) in the P2P group and 94 participants (38.6%) in the SCS group visiting a UC clinic or ED and/or being hospitalized at least once over the 12 months of the study (Figure 2). Among the randomly selected 10% of participants who reported using medical care for whom we compared their self-reported use to what was documented in their medical record, there was a 100% match between what was reported and documented (data not shown).

After propensity score adjustment (Table 3), we found a statistically significant higher rate of hospitalization in the P2P group than the SCS group (0.68 hospitalization per year vs 0.44 hospitalization per year; RR, 1.54; 95% CI, 1.14-2.07; *P* = .005). This finding appears to be associated with higher rates of hospitalization in the P2P group compared with the SCS at the West Palm Beach (0.83 hospitalization per year vs 0.53 hospitalization per year) and Rochester (0.61 hospitalization per year vs 0.35 hospitalization per year) sites. There were no significant differences between the 2 groups in the rates of ED visits (RR, 0.98; 95% CI, 0.73-1.31; *P* = .87), UC visits (RR, 0.79; 95% CI, 0.47-1.32; *P* = .37), or composite health care utilization (RR, 1.21; 95% CI, 0.97-1.52; *P* = .10).

**Table 3.** Estimated Rates of Health Care Utilization and RRs of Reporting for UC and ED Visits and Hospitalizations Over 12 Months by Group^a^

Type of health care use	Overall			Los Angeles, CA			Rochester, NY			West Palm Beach, FL		
	Group		RR (95% CI)	Group		RR (95% CI)	Group		RR (95% CI)	Group		RR (95% CI)
	SCS (n = 224)	P2P (n = 213)		SCS (n = 38)	P2P (n = 35)		SCS (n = 97)	P2P (n = 95)		SCS (n = 89)	P2P (n = 83)	
UC visit	0.16 (0.11-0.22)	0.12 (0.08-0.19)	0.79 (0.47-1.32)	0.35 (0.24-0.51)	0.16 (0.08-0.32)	0.46 (0.21-1.01)	0.08 (0.04-0.16)	0.08 (0.04-0.19)	1.03 (0.36-2.97)	0.13 (0.07-0.26)	0.12 (0.05-0.25)	0.87 (0.32-2.41)
ED visit	0.28 (0.23-0.34)	0.27 (0.22-0.35)	0.98 (0.73-1.31)	0.36 (0.24-0.54)	0.26 (0.14-0.48)	0.73 (0.35-1.51)	0.38 (0.30-0.48)	0.34 (0.25-0.47)	0.90 (0.60-1.35)	0.15 (0.10-0.23)	0.22 (0.15-0.31)	1.40 (0.82-2.40)
Hospitalization	0.44 (0.36-0.54)	0.68 (0.54-0.85)	1.54 (1.14-2.07)^b^	0.49 (0.30-0.79)	0.52 (0.28-0.96)	1.07 (0.49-2.34)	0.35 (0.25-0.49)	0.61 (0.42-0.88)	1.73 (1.05-2.85)^b^	0.53 (0.39-0.71)	0.83 (0.62-1.12)	1.57 (1.03-2.40)^b^
Primary composite measure	0.91 (0.78-1.06)	1.10 (0.92-1.31)	1.21 (0.97-1.52)	1.20 (0.78-1.86)	0.92 (0.51-1.63)	0.76 (0.37-1.57)	0.81 (0.65-1.00)	1.02 (0.79-1.32)	1.26 (0.90-1.77)	0.81 (0.65-1.02)	1.16 (0.93-1.45)	1.43 (1.04-1.96)^b^

Abbreviations: ED, emergency department; P2P, peer-to-peer per group; RR, risk ratio; SCS, standard community services; UC, urgent care.

^a^ Rates were estimated using negative binomial regression analysis with inverse propensity score weighting.

^b^ *P* < .05.

## Discussion

In this population of community-dwelling older adults, the P2P group had a significantly higher rate of hospitalization than the SCS group. There were no significant differences between the groups on the other measures. Our findings did not support our hypothesis that there would be less use of health care in the group receiving P2P support and contrast with those of other studies.

The studies^25,26,27,28,29,30,31^ we identified as measuring the impact of peer support were small, had limited follow-up, or were conducted outside the US. Three studies investigated the impact of a volunteer visiting program to older adults and measured changes over a 6-week period.^26,27^ Together they all found statistically significant improvements in self-reported measures of life satisfaction, overall quality of life, health, and social support in older adults receiving peer support. One randomized clinical trial^28^ conducted in the Republic of Korea demonstrated that a volunteer peer support program provided to half the older adults living alone (47 participants in total, including 26 in the intervention group) and followed over 12 months had significantly higher scores in physical and general health than the control group; in addition, depression lessened and social functioning and satisfaction with social support improved significantly in the intervention group compared with the control group.

To our knowledge, this is the first longitudinal study of the role that P2P support plays in promoting aging in place in the US. We are not sure why we did not find an association between P2P support and health care utilization. Our P2P group was frailer at the beginning of our study than the control group, and this likely affected our ability to fully measure the outcomes associated with P2P support. Data from our qualitative interview study with the older adults who received P2P support suggest that the service and relationships they developed through the program were critical to their wellness, suggesting that our design or sample size were not robust enough to effectively measure the impact of P2P support.^32^ It is possible that the association of P2P support with hospitalizations may have occurred because the peers, in general, helped the older adults they supported to discuss what to do when they were not feeling well, to call their physicians, and get guidance that led to hospitalization.

### Limitations

Our study was limited by the facts that we could not randomize participants and that our study groups differed significantly on some important baseline characteristics, including income distribution. In addition, 78% of our sample identified as White, limiting generalizability to minority populations. However, given the multisite design of our study and the fact that we studied a real-world program, our findings are generalizable to urban communities in the US that have strong aging support services. Second, although we asked about the use of peer services, the tool we used was not good enough to sort out how much peer support each person in the P2P group received. As a result, we could not look at the “dose” of peer support as we had planned. Third, although we anticipated that 12 months would be too short a period for community-dwelling older adults to decline to the point that they would need to move into a nursing home, we did think that 12 months would be sufficient to see differences in health care utilization. In fact, the follow-up period may have been too short to see these differences. This latter view was borne out in our study because very few of our participants transitioned to a nursing home or other higher level of care (data not shown). Furthermore, our health care utilization data was self-reported; although our validation process suggests that these data were reliable, it is possible that some visits were not reported.

## Conclusions

Given the aging of the US population and the fact that most older adults want to live and age in their communities, we need to study what interventions effectively support aging in place. Qualitative data we collected at the end of our study suggest that community-based P2P support programs promote aging in place and are of value to older adults, their families, and the peer volunteers who provide that support.^32^ We need more research on these types of inexpensive, pragmatic interventions, including how peer support advances aging in place in rural and more diverse aging communities and among older adults who would likely benefit the most, including those aged 80 years and older or who are more socially isolated. We also suggest that investigators explore what is already happening in communities to promote aging in place. At the time we applied for Patient Centered Outcomes Research Institute funding, we found that P2P support programs have been around a long time, but that very little research had been done on whether and how these programs support aging in place, despite their high face validity. Similar programs should be evaluated in randomized clinical trials. In addition, to understand fully how these support programs affect nursing home placement, studies would need to have a longer follow-up period and would need to focus on an older population. Finally, future research should attempt to capture the frequency with which participants engages with their peers and the frequency with which they use SCSs.

## Notes

Supplement. **eTable.** Variables Included in the Propensity Score
