# Supplementary material for: Evaluation of Peer-to-Peer Support and Health Care Utilization Among Community-Dwelling Older Adults
Source: JAMA Netw Open. 2020 Dec 15;3(12):e2030090. doi: 10.1001/jamanetworkopen.2020.30090 (PMC7739125; doi:10.1001/jamanetworkopen.2020.30090)
Supplement: Supplement. — eTable. Variables Included in the Propensity Score [file jamanetwopen-e2030090-s001.pdf]

Supplemental Online Content

Jacobs EA, Schwei R, Hetzel S, et al. Evaluation of peer-to-peer support and health care utilization among community-dwelling older adults. *JAMA Netw Open*. 2020;3(12):e2030090. doi:10.1001/jamanetworkopen.2020.30090

eTable. Variables Included in the Propensity Score

This supplemental material has been provided by the authors to give readers additional information about their work.

© 2020 Jacobs EA et al. *JAMA Network Open.*

eTable. Variables Included in the Propensity Score

Category	Variable Description	Variable Format
Demographic	Age	Continuous
	Do you live alone?	Dichotomous
	Do you receive in home care from someone other than your spouse?	Dichotomous
	Have you previously been in a nursing home?	Dichotomous
	How many times have you seen a doctor in the last 6 months?	Continuous
	I drive myself to get around	Dichotomous
	A family member drives me to get around	Dichotomous
	A friend or helper drives me to get around	Dichotomous
	I use other ways to get around	Dichotomous
	Have you driven in the last 3 months	Dichotomous
	Did anyone in your immediate family drive a car in the last 3 months?	Dichotomous
Health condition	Total number of medical conditions (0 - 6)	Continuous
	General Self Efficacy questionnaire total score	Continuous
	Loneliness questionnaire total score	Continuous
	CESD depression symptoms questionnaire total score	Continuous
	Anxious classification based on Geriatric Anxiety Index total > 2	Dichotomous
	How often do you get out of your house?	5 response ordinal scale
	How often are you able to go where you would like to go?	3 response ordinal scale
	Is getting where you need to go usually a problem?	3 response ordinal scale
	Have you ever stayed in bed most of the time over the last 2 weeks?	Dichotomous
	RAPA - Aerobic physicality status	5 response ordinal scale
	RAPA - Strength status	3 response ordinal scale
	ADL - Disability status	3 response ordinal scale
	IADL - Disability total score	Continuous
	Rosow-Breslow - physical ability total score	Continuous
	NAGI - total score about physical ability	Continuous
	SF-12 - Mental composite score	Continuous
	SF-12 - Physical composite score	Continuous
	Brief Resilience Scale total	Continuous
	Overall health screening score	Continuous

© 2020 Jacobs EA et al. *JAMA Network Open.*
